# Case Report: Successful Management and Long-Term Follow-Up of Bilateral Ureteral Ligation in a Dog Secondary to Cryptorchid Castration Utilizing Bilateral Ureteral Stents and a Neoureterocystostomy Procedure

**DOI:** 10.3389/fvets.2022.903638

**Published:** 2022-06-02

**Authors:** Rebecca Walton, Megan Mickelson, Jean-Sebastien Palerme, April Blong, Meredith 't Hoen

**Affiliations:** ^1^Department of Clinical Sciences, Iowa State University, Ames, IA, United States; ^2^Department of Clinical Sciences, University of Missouri, Columbia, KY, United States

**Keywords:** acute kidney disease, ureteral, castration, cryptorchid surgery, ureteral stents

## Abstract

**Objective:**

To report the successful management of the bilateral ureteral obstruction secondary to ureteral ligation during unilateral cryptorchid surgery in a dog with the utilization of bilateral ureteral stent placement and a neoureterocystostomy procedure.

**Summary:**

A 7-month-old male-castrated Weimaraner weighing 30 kg was presented to a university teaching hospital for evaluation of a 4-day history of lethargy, vomiting, and stranguria following exploratory laparotomy for a left-sided unilateral cryptorchid castration. Based on the concurrent presence of severe azotemia and ultrasonographic findings of bilateral hydronephrosis and hydroureter, the dog was diagnosed with the suspected bilateral ureteral obstruction. The dog underwent a laparotomy which revealed bilateral ureteral ligation which was corrected with a left-sided neoureterocystostomy and right-sided retrograde ureteral stent placement. Subsequent placement of a left-sided ureteral stent due to complications with the neoureterocystostomy site was performed later. Ultimately, both ureteral stents were able to be removed a few months later. The dog was clinically doing well with a baseline creatinine of 1.5 mg/dl (132.6 μmol/L) 532 days following initial bilateral ureteral ligation.

**New or Unique Information Provided:**

This case report describes the successful long-term management of iatrogenic bilateral ureteral obstructions in a male dog using a combination of bilateral ureteral stents and neoureterocystostomy.

## Introduction

Ureteral obstructions pose both diagnostic and therapeutic challenges and can be secondary to a variety of etiologies (1). Ureteral obstruction most commonly result from ureterolithiasis; however, other causes of ureteral obstructions have been reported including ureteral strictures, blood clots, or neoplasia (1–5). Ureteral injuries and obstructions secondary to iatrogenic causes have been described, most commonly secondary to ovariohysterectomy in both dogs and cats (6, 7). Treatment of ureteral injuries and obstructions can vary and include both medical management and surgical interventions which encompass both traditional and interventional techniques with varying success rates. Traditional techniques include ureterotomy, ureteral reimplantation with or without renal descensus, or ureteronephrectomy, while interventional techniques described include ureteral stenting or subcutaneous ureteral bypass (SUB) placement (2, 6, 8). Short-term success rates in benign ureteral obstruction of up to 100% have been described with interventional techniques (9).

Despite descriptions of ureteral injury during ovariohysterectomy and the resulting treatment and outcomes, there are no reports of ureteral obstruction secondary to cryptorchid castration in a dog. This case report describes the successful management of bilateral ureteral ligation in a dog using the combination of medical management and surgical interventions including neoureterocystostomy and bilateral ureteral stenting.

## Case Report

**Figure 1:**
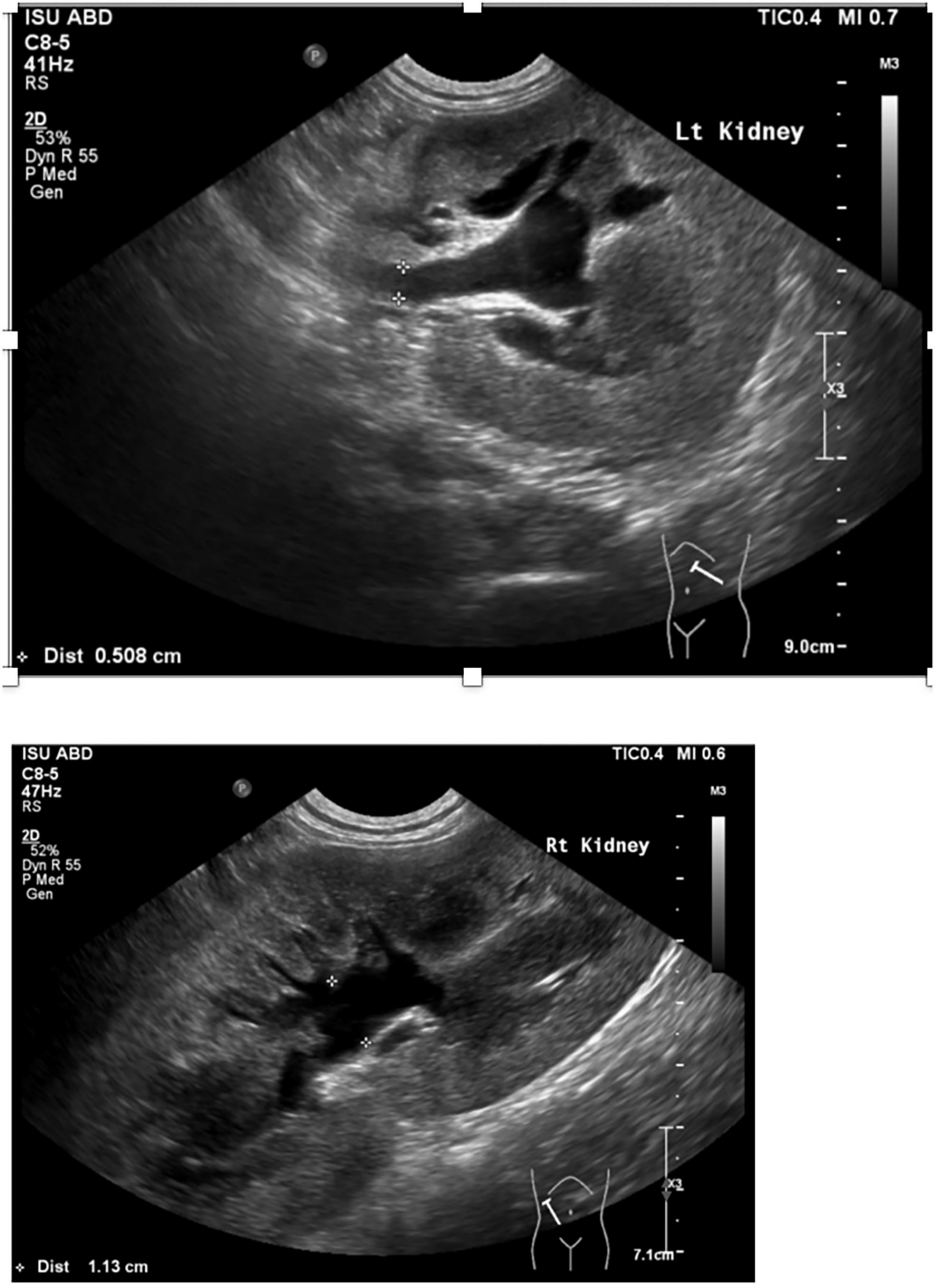
Abdominal ultrasound image of left and right kidney and proximal ureter including pelvic dilation and ureteral dilation.

A 7-month-old male castrated Weimaraner presented to a university teaching hospital for evaluation of a 4-day history of lethargy, vomiting, and stranguria with reported anuria (day 0). The dog underwent an exploratory laparotomy 4 days prior to presentation for a unilateral cryptorchid castration. During cryptorchid surgery, a ventral midline incision was made cranial to the prepuce and the right testicle was identified in the retroperitoneal space. The right testicle was partially exteriorized, double ligated *in situ* with 2-0 absorbable suture and removed. The abdomen was closed without complication and a prescrotal incision was made for the left testicle, which was exteriorized, clamped, and ligated with 3-0 absorbable suture.^1^ No complications were noted throughout the perioperative and immediate postoperative period and the dog was discharged the day of surgery with carprofen and cefpodoxime (both 5 mg/kg orally once a day for 7 days). Following discharge, the dog was lethargic, uncomfortable, and vomited once. He was noted to be unsuccessfully straining to urinate. On presentation, the dog's vital signs were within normal limits. Abdominal palpation revealed a painful abdomen with markedly enlarged and painful kidneys. Initial diagnostics included a venous blood gas, chemistry profile, and focused assessment with sonography for trauma/tracking/triage (FAST) scan. Venous blood gas revealed a metabolic acidosis, pH 7.310 (RI 7.31–7.42), CO_2_ 41.5 mmHg (RI 35–45 mmHg), HCO_3_ 20.4 mmol/L (RI 17–24 mmol/L), BE −5.8 (RI −4 to 4) and hyperkalemia, 6.01 mEq/L (RI 3.9–5.3 mEq/L). Chemistry profile revealed severe azotemia, BUN 166 mg/dl, 59.3 mmol/L (RI 10–30 mg/dl; 3.2–10.4 mmol/L), creatinine 15.4 mg/dl, 1,361 μmol/L (RI, 0.5–1.8 mg/dl; 35–124 μmol/L), and a hyperphosphatemia of 15.2 mg/dl, 4.9 mmol/L (RI 3.2–6.0 mg/dl; 0.61–1.61 mmol/L) (Table 1). The FAST scan revealed scant peritoneal effusion. The dog was admitted to the hospital after hours and imaging was not available till the following morning. That night the dog was hospitalized with intravenous balanced isotonic crystalloid fluids (see text footnote 1) at a dose of 40 ml/kg/day in addition to 5% dehydration replaced over 6 h, ampicillin–sulbactam^2^ (30 mg/kg IV q24h), hydromorphone^3^ (0.1 mg/kg IV q6h), maropitant^4^ (1 mg/kg IV q24h), and trazodone^5^ (3.3 mg/kg PO as needed, q8h). Urine production, assessed by indwelling urinary catheter, was negative for the following 8 h. In total, 12 h post admission to the intensive care unit (day 1), an abdominal ultrasound was performed, revealing bilateral hydronephrosis (right renal pelvis = 11 mm, left renal pelvis = 10 mm) and hydroureter (Figure 1). A curvilinear shadow was noted at the tapering end of the left ureter and a mild amount of abdominal effusion. Complete blood cell count and repeat chemistry profile revealed a potassium of 8.0 mEq/L (RI 3.9–5.3 mEq/L), BUN 163 mg/dl, 58.2 mmol/L (RI 10–30 mg/dl; 3.2–10.4 mmol/L), creatinine 16.8 mg/dl,1,485 μmol/L (RI, 0.5–1.8 mg/dl; 35–124 μmol/L) and phosphorus of 13.4 mg/dl, 4.3 mmol/L (RI 3.2–6.0 mg/dl; 0.61–1.61 mmol/L). Biochemical analysis of peritoneal effusion revealed a BUN of 174 mg/dl and creatinine of 17.4 mg/dl, which was not consistent with uroabdomen. Hyperkalemia was treated prior to general anesthesia with 10% calcium gluconate^6^ (1 ml/kg IV diluted 1:4), 50% dextrose^7^ (0.5 ml/kg diluted 1:4) and regular insulin^8^ (0.2 U/kg IV).

**Table 1:** Serial biochemistry results from the selected dates.

	**Day 0**	**Day 1**	**Day 2**	**Day 3**	**Day 4**	**Day 5**	**Day 6**	**Day 9**	**Day 10**	**Day 27**	**Day 34**	**Day 72**	**Day 529**
Sodium (mEq/L)	158	142	150	144	140	139	137	141	141	140	139	139	
Potassium (mEq/L)	6.4	8.0	4.2	4.4	4.2	4.9	4.5	3.7	3.9	5.4	4.1	5.5	
Phosphorus (mg/dL)	15.2	13.4	7.8	5.5	4.6	4.2	5.3	5	5.1	7.3	6.7	5.4	
Magnesium (mg/dL; mmol/L)		2.02; 0.83	1.76; 0.72	1.67; 0.69	1.75; 0.72		1.48; 0.61					2.0; 0.82	
BUN (mg/dL; mmol/L)	166; 59.3	163; 58.2	69; 24.6	31; 11	18; 6.4	13; 4.6	14; 5	12; 4.2	11; 3.9	20; 7.1	13; 4.6	12; 4.2	14; 5
Creatinine (mg/dL; μmol/L)	15.4; 1,362	16.8; 1,432	4.9; 433	1.9; 168	1.5; 136	1.2; 106	1.2; 106	1.7; 150	1.4; 124	1.0; 88	1.1; 97	1.7; 150	1.3; 11
Albumin (g/dL; mg/dL)	2.9; 290	2.7; 270	2.0; 200	1.8; 180	1.7; 170	1.6; 160	1.7; 170	2.3; 230	2.4; 240	3.4; 340	2.4; 240	2.9; 290	3.0; 300

*Day 0 is the day of presentation to the university teaching hospital*.

**Figure 2:**
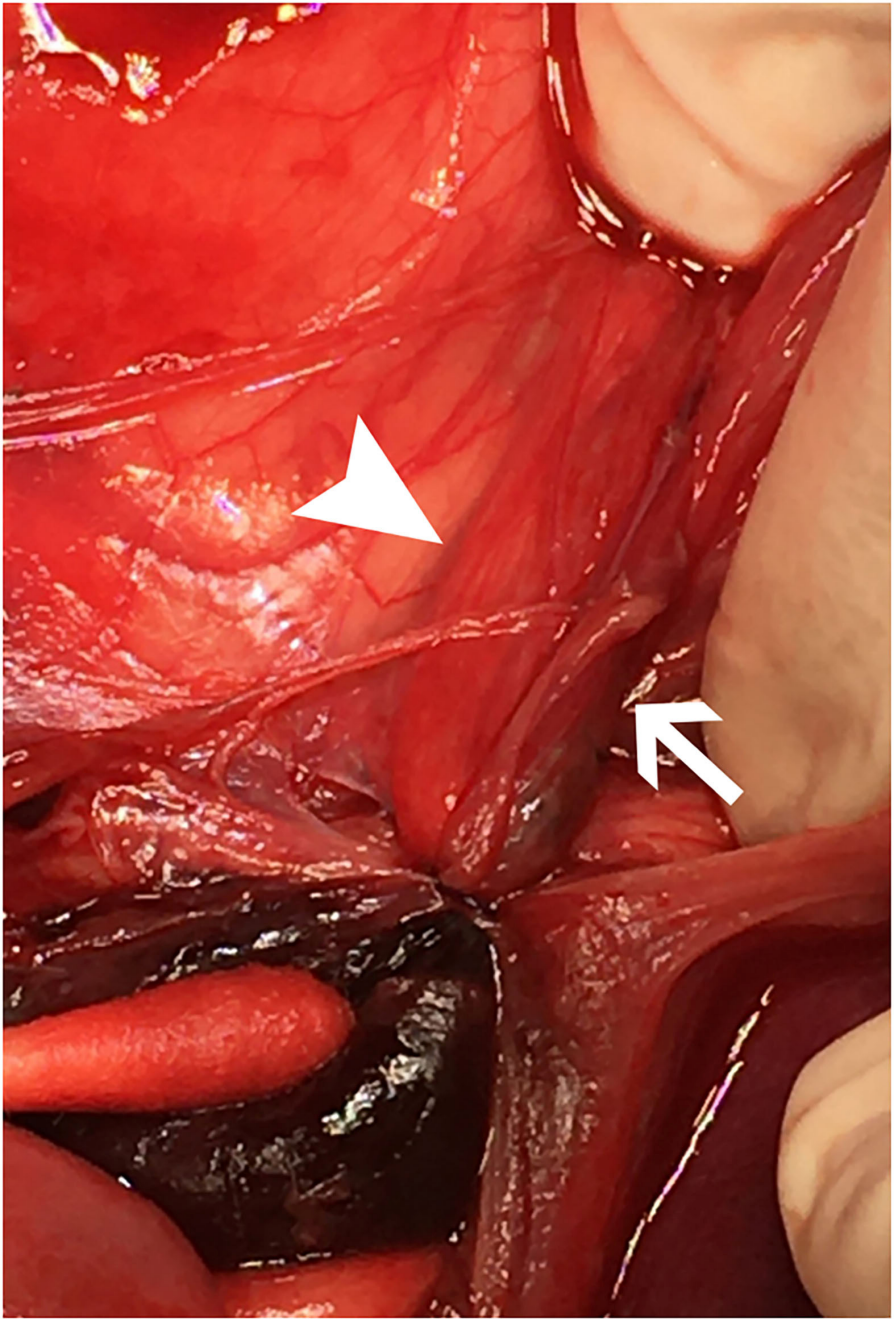
Cranial is at the top. The image shows a close-up view of the sutures, intact, surrounding both ureters within the left retroperitoneal space immediately upon entry into the abdomen, prior to dissection. The right ureter (white arrowhead) and left ureter (white arrow) are both entrapped with a hematoma caudal to the ligatures with a cotton-tipped applicator pointing to it in the lower left of the image.

**Figure 3:**
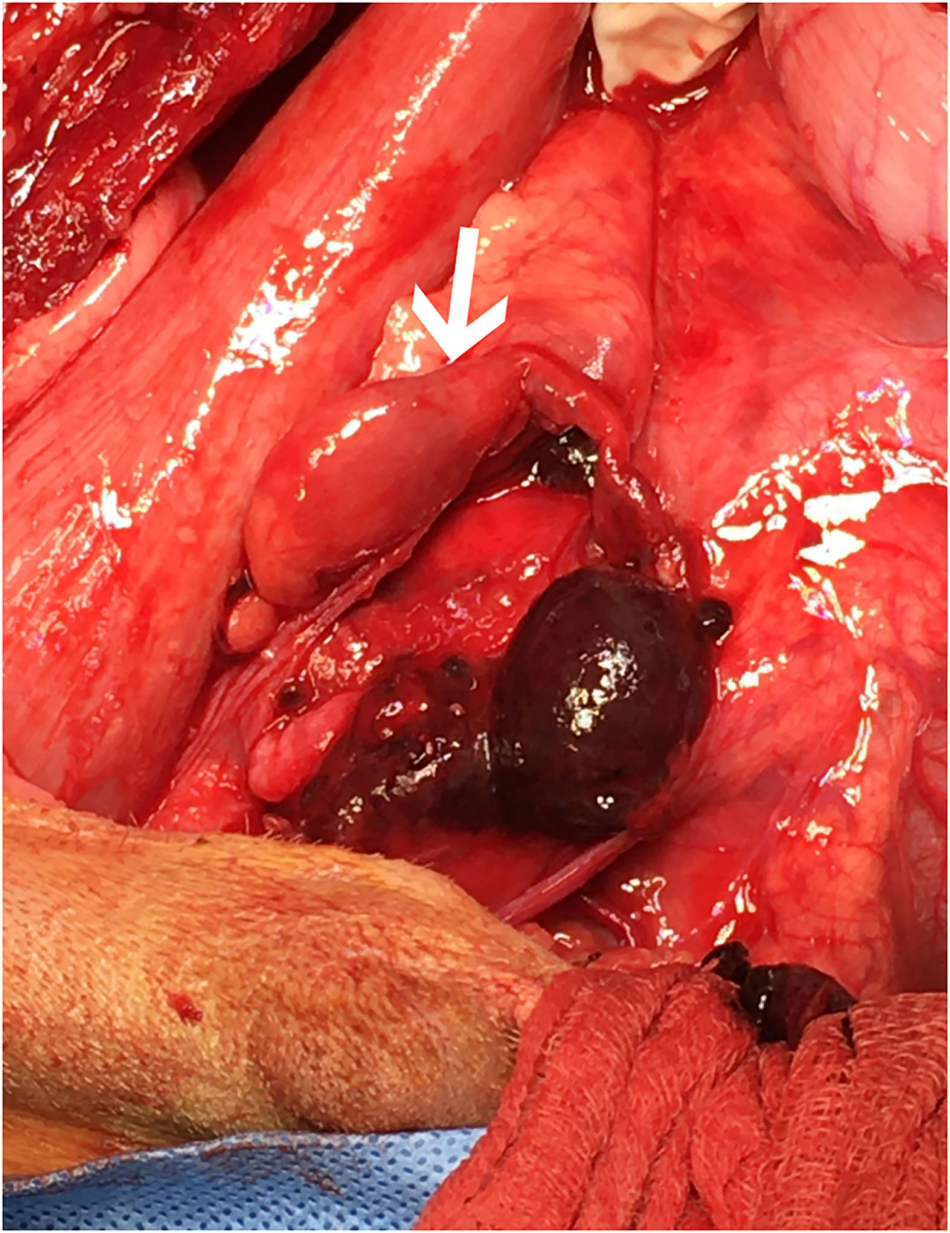
Cranial is to the left. The surgeon is retracting the descending colon to the patient's left side to demonstrate the region of interest following dissection and removal of the encircling ligatures. The proximal end of the left ureter (white arrow) is shown with significant dilation. Following dissection, the proximal and distal ends of the left ureter were found to be completely separate within the large hematoma shown.

The dog underwent abdominal exploratory surgery, where the bladder was assessed to be empty, the kidneys were bilaterally enlarged and turgid with diffuse capsular angiogenesis, and both ureters were noted to enter the bladder at the dorsal aspect of the trigone. Both the ureters were tracked cranially where multiple sutures were found to be encircling both ureters in combination with the right vas deferens (Figures 2, 3). The right ureter was freed *via* removal of the encircling ligature (Figure 2) and three additional sutures were dissected and removed to free the left ureter. A cystotomy was performed on the ventral aspect of the bladder. The left ureter was strictured and found to be non-patent based on the inability to pass a 0.025″guidewire retrograde from the cystotomy incision past the area of concern, with additional evidence of partial transection (Figure 3). The transected portion of the left ureter was passed through the bladder, the strictured end was debrided using tenotomy scissors and the ureter was spatulated. A neoureterocystostomy with 6-0 monofilament suture with a taper point needle (Monocryl^9^), in a simple interrupted pattern, was performed utilizing an intravesicular technique at the apex of the bladder, with the appropriate flow at the neoureterocystostomy site. Urine was noted to flow into the right ureterovesicular junction; however, the ureter remained strictured focally where the suture had been present. Consequently, a 4.7 French 20 cm ureteral stent^10^ was placed in a retrograde manner over a 0.025″ guidewire and passed into the right renal pelvis under fluoroscopic guidance. The guidewire was removed and the stent was confirmed to be placed correctly within the right renal pelvis using fluoroscopy. The bladder was closed in a routine fashion and a cystopexy was performed to alleviate tension on the reimplantation site. The abdomen was lavaged with warm fluids and a gastrostomy tube and closed suction abdominal drain^11^ were placed prior to abdominal closure. An eight French urinary catheter^12^ and a seven French triple lumen jugular catheter^13^ were also placed prior to recovery. The dog was hospitalized in the intensive care unit on constant rate infusions of fentanyl^14^ (3–5 mcg/kg/h), ketamine^15^ (0.3–0.5 mg/kg/h), lidocaine^16^ (20–50 mcg/kg/min) and a balanced isotonic crystalloid (see text footnote 1) (292 ml/kg/day) in addition to the previous treatments. Urine output was monitored and fluid administration was adjusted accordingly every 6 h. Chemistry profile 1 day postoperatively (day 2) revealed marked improvement in the azotemia, BUN 69 mg/dl, 24 mmol/L (RI 10–30 mg/dl; 3.2–10.4 mmol/L), creatinine 4.9 mg/dl, 433 μmol/L (RI, 0.5–1.8 mg/dl; 35–124 μmol/L), phosphorus 7.8 mg/dl, 2.5 mmol/L (RI 3.2–6.0 mg/dl; 0.61–1.61 mmol/L) and a moderate hypoalbuminemia was noted, albumin 2.0 mg/dl, 20 g/L (RI 2.7–4.0 mg/dl; 25–40 g/L) (Table 1). Day 2 through day 8, the dog remained hospitalized for supportive care and the following treatments were added: ondansetron^17^ (1 mg/kg IV q8h), cisapride^18^ (1 mg/kg PO q8h), gabapentin^19^ (10 mg/kg PO q8h), pantoprazole^20^ (1 mg/kg IV q12h), and magnesium sulfate^21^ supplementation (0.3 mEq/kg) as needed based on bloodwork findings. The dog was provided 100% resting energy requirement (RER) per day *via* a feeding tube. The closed suction abdominal drain fluid was quantified and emptied every 6 h for 4 days postoperatively. The urinary catheter remained in place with urinary catheter care and urine output monitoring for 2 days postoperatively. Urine output for the first 48 h following surgery was ~200 ml/h (6.5 ml/kg/h) and the urinary catheter was removed 2 days post operatively. On day 6 of hospitalization, the abdominal drain production increased and cytology was consistent with marked neutrophilic inflammation with no intracellular bacteria seen; however, enrofloxacin^22^ (14 mg/kg PO once a day) was added. Biochemical values and abdominal fluid were static throughout days 6–11 of hospitalization and the dog was clinically stable. On day 11 of hospitalization, the dog's azotemia progressed and abdominal ultrasound revealed marked, progressive left-sided hydronephrosis (left renal pelvis = 22 mm), hydroureter, and a distal ureteral obstruction with improved right-sided hydronephrosis and appropriately placed right-sided ureteral stent. Cystoscopy was performed in an attempt to further evaluate the left ureteral reimplantation site and multiple attempts to cannulate it with a 0.025″ hydrophilic guidewire failed so laparotomy was pursued. During the initial abdominal approach, a pocket of turbid fluid, suspected to be a body wall abscess located at the distal end of the abdominal incision, was found and sampled for culture. Significant peritonitis and multiple adhesions between the viscera and abdominal wall were present. The spleen was adhered to the left kidney, requiring a hilar splenectomy for visualization. A large fibrous pocket containing serosanguinous fluid was present caudal to the left kidney in the retroperitoneal space where the prior cryptorchid ureteral ligation had been performed. The left kidney and ureter were noted to be significantly enlarged. The obstructed left ureter was bluntly dissected, a ventral cystotomy incision was made. The obstructed left ureter was isolated *via* blunt dissection at the implantation site and the implantation site at the level of the urinary bladder was excised. The cystotomy site was extended caudally and a 3.5 French red rubber catheter (w) was passed through the ureter in a retrograde manner, which confirmed obstruction from the surrounding adhesions and suture granuloma at the reimplantation site. The distal ureter was reimplanted 1.5 cm to the right of the apex of the bladder. The distal ureter was grasped, pulled into the bladder, and the bladder wall was everted manually with the use of stay sutures, the distal ureter was spatulated with tenotomy scissors and a 5 French red rubber catheter^23^ was kept in place within the ureter to aid in anastomosis. The neoureterocystostomy was performed with a simple interrupted suture pattern using 6-0 monofilament suture with a taper point needle [Monocryl (see text footnote 9)]. A 0.035 mm guidewire was passed retrograde utilizing fluoroscopy into the renal pelvis and a ureteral measure catheter was temporarily placed over the guidewire to guide stent size. A 4.7 French 20 cm ureteral stent (see text footnote 10) was then placed in the left ureter in a retrograde manner over the guidewire. A closed suction abdominal drain (see text footnote 11) was placed. The dog recovered in the intensive care unit on balanced isotonic crystalloid fluids (see text footnote 1), fentanyl (see text footnote 14) (3–5 mcg/kg/h), ketamine (see text footnote 15) (0.3–0.5 mg/kg/h), and norepinephrine^24^ (0.1 mcg/kg/min for 6 h) in the postoperative period. The dog was maintained on enrofloxacin (see text footnote 22) (14 mg/kg PO q24h), trazodone (see text footnote 5) (6.5 mg/kg PO q8h), gabapentin (see text footnote 19) (10 mg/kg PO q8h), and chloramphenicol^25^ (43 mg/kg PO q8h for 7 days) pending culture, which had heavy growth of *Escherichia coli* that was susceptible to amikacin, gentamicin, imipenem, and intermediate susceptibility to chloramphenicol. The dog recovered uneventfully and was hospitalized for 14 days following the second abdominal exploratory, totaling 28 days of hospitalization. The dog was hospitalized for 14 days following the second procedure in order to monitor biochemical values and taper fluid support. The dog was ultimately discharged with a creatinine of 1.0 mg/dl, 88 μmol/L (RI, 0.5–1.8 mg/dl; 35–124 μmol/L) (Table 1) on trazodone (see text footnote 5) (6.5 mg/kg PO q8h) and supplemented enteral water 36 ml/kg/day *via* feeding tube. The dog was re-evaluated 4 days following discharge (day 32 from initial presentation). At that time, the dog was doing well clinically and eating RER by mouth. His creatinine was 1.2 mg/dl, 106 μmol/L (RI, 0.5–1.8 mg/dl; 35–124 μmol/L) (Table 1) and he was continued on oral water with no medications. On day 35, he was evaluated for a 1-day history of lethargy and decreased appetite. On presentation, he was febrile (103.7 F) with moderate pain on abdominal palpation. Diagnostics revealed static creatinine and urinalysis demonstrated significant pyuria and the dog was started on trimethoprim/sulfamethoxazole (SMZ–TMP; 32 mg/kg PO q12)^26^ pending culture results. Urine culture had heavy growth of *Escherichia coli*, sensitive to SMZ–TMP (see text footnote 26). Radiographs revealed the bilateral ureteral stents to be appropriately placed with no other abnormalities noted. The dog was discharged on day 40, after 5 days of intravenous crystalloid fluids, on SMZ–TMP (see text footnote 26) alone. The dog was evaluated on day 52 where his creatinine was stable at 1.1 mg/dl, 97 μmol/L (RI, 0.5–1.8 mg/dl; 35–124 μmol/L) (Table 1). Urine culture performed at that visit showed low growth of *E.coli* so SMZ–TMP (see text footnote 26) was continued. The dog was evaluated 14 days later, day 66 overall, for a routine recheck and was doing clinically well at that time. Urine culture was negative and chemistry revealed a hyponatremia 139 mEq/L (144–154 mEq/L), hyperkalemia 5.5 mEq/L (RI 3.9–5.3 mEq/L) and creatinine of 1.7 mg/dl, 150 μmol/L (RI, 0.5–1.8 mg/dl; 35–124 μmol/L). All the medications were discontinued at this time. The gastrostomy tube was in place for 64 days to supplement water and nutrition as necessary and removed uneventfully. Despite the increase in baseline creatinine, the dog continued to do well at home. In total, 72 days from the date of presentation, the dog had both ureteral stents cystoscopically removed using a 1.9 French 120 cm basket device^27^ without complication. The dog was hospitalized for 4 days following stent removal to ensure no acute kidney injury or complications developed following stent removal. Creatinine was 1.6 mg/dl, 141 μmol/L (RI, 0.5–1.8 mg/dl; 35–124 μmol/L) at the time of discharge without any ongoing medications. The dog was clinically doing well with a baseline creatinine of 1.5 mg/dl (RI, 0.5–1.8 mg/dl; 35–124 μmol/L) 529 days later (532 days post bilateral ureteral ligation).

## Discussion

Despite literature evaluating therapeutic interventions and outcome in dogs and cats experiencing iatrogenic ureteral ligation and trauma during ovariohysterectomy, there are no reports describing this complication in patients undergoing cryptorchid neuter. Ureteral obstructions can result from intraluminal obstruction, mural lesions, or extraluminal compression (10). Ureteral obstruction results in significant renal effects as the increase in ureteral pressure results in a decrease in the renal blood flow to 40% of normal within the first 24 h and 20% of normal by 2 weeks (1, 11, 12). These changes to renal blood flow result in decreased glomerular filtration rate, which may result in irreversible renal damage (1, 11). Clinical signs of ureteral obstruction vary depending on underlying renal function and unilateral vs. bilateral ureteral involvement. Bilateral ureteral involvement has been noted in up to 12.5% of dogs and 19% of cats with urolithiasis and 37% of dogs and cats with ureteral complications secondary to ovariohysterectomy (4, 6). Clinical signs associated with ureteral obstructions include abnormalities in urination, persistent urinary tract infections, abdominal pain, or systemic signs including vomiting, anorexia, or lethargy (6, 10). The onset of the clinical signs in cases of iatrogenic ligation is variable, with 58% developing signs at anesthetic recovery and 32% displaying delayed clinical signs, ranging from 1 to 16 days postoperatively (6). Diagnosis of ureteral obstructions can be made with abdominal imaging such as abdominal ultrasound, percutaneous antegrade pyelography or computed tomography (10, 13). Treatment of ureteral obstructions varies, depending on the underlying etiology. Generally, treatment includes a combination of medical management to allow for patient stabilization and surgical intervention. Medical stabilization often involves combination therapy of intravenous fluids, osmotic diuretics, and smooth muscle relaxants, often being instituted immediately to aid in-patient stabilization (14). While medical management is essential in-patient stabilization, medical management alone has a very poor success rate in cats and dogs, surgical interventions remain the standard of care (2, 4, 15, 16). Surgical interventions include traditional management or interventional techniques. Traditional surgical interventions vary based upon the underlying cause and surgeon preference and include ureterotomy, ureteral reimplantation with or without renal descensus, or ureteronephrectomy (2). Newer interventional techniques may also be employed in the management of ureteral obstructions which include placement of percutaneous nephrostomy tubes, ureteral stents, or subcutaneous ureteral bypasses (2, 8). Ureteral stenting is a relatively common interventional technique that allows for bypassing the obstruction and passive ureteral dilation and can be a viable long-term solution, remaining in place for years (2, 17). Ureteral stenting in dogs secondary to benign etiologies including ureterolithiasis, stricture, or a combination of the two has a reported success rate of 98% survival to discharge and no reported deaths associated with complications of the procedure, renal failure, or recurrent obstructions (18).

In addition to descriptions of traditional and interventional techniques utilized in the management of ureteral obstruction, several case reports and a recent retrospective case series describe successful management of ureteral obstruction and injuries associated with ovariohysterectomy in both the dogs and cats (6, 7, 19). Surgical techniques utilized in the management of iatrogenic ureteral ligation and trauma include ureteroneocystostomy with or without renal descensus, ureteronephrectomy, subcutaneous ureteral bypass, and ureteral stents (6, 7, 19, 20). Outcomes for these cases were considered excellent in 68% of cases, good in 5% of cases, and poor in 22% of cases (6).

In this case, bilateral ureteral ligation during cryptorchid neuter was successfully managed using a combination of traditional and interventional surgical techniques and medical management. Neoureterocystostomy was initially performed for the fully damaged left ureter without stent placement in an attempt to reduce the number of implants and the need for future implant removal in this juvenile dog due to potential risks of recurrent and resistant urinary tract infections, encrustation, and need for additional procedures for adjusting implants (1, 2). Based on the severity of stricture in the right ureter, a ureteral stent was placed during the initial surgery. Unfortunately, stenosis of the left neoureterocystostomy site developed 4 days postoperatively, thought to be related to tension and granuloma formation causing extramural compression. The stenosis of the left ureter was subsequently treated with second ureteral reimplantation and a temporary ureteral stent. Ureteral implants are associated with a risk of infection, which was documented in this dog with multiple positive urine cultures for resistant strains of *Escherichia coli*. Due to the recurrent urinary tract infections and the static creatinine, the decision was made to remove the stents 70 days post placement. Overall, multiple techniques including surgical and interventional strategies to manage the bilateral ureteral obstruction and stenosis were utilized, all of which resulted in a successful long-term outcome.

## Data Availability Statement

The original contributions presented in the study are included in the article/supplementary material, further inquiries can be directed to the corresponding author/s.

## Ethics Statement

Ethical review and approval was not required for the animal study because this is a case report and therefore not reviewed by animal ethics committee. Written informed consent was obtained from the owners for the participation of their animals in this study.

## Author Contributions

RW, MM, J-SP, AB, and MH contributed to manuscript writing and editing. All authors contributed to the article and approved the submitted version.

## Conflict of Interest

The authors declare that the research was conducted in the absence of any commercial or financial relationships that could be construed as a potential conflict of interest.

## Publisher's Note

All claims expressed in this article are solely those of the authors and do not necessarily represent those of their affiliated organizations, or those of the publisher, the editors and the reviewers. Any product that may be evaluated in this article, or claim that may be made by its manufacturer, is not guaranteed or endorsed by the publisher.

## Footnotes

^1^Lactated ringers, B. Braun, Irvine, CA.

^2^Ampicillin-sulbactam, Auromedics Pharma LLC, E. Windsor, NJ.

^3^Hydromorphone, Hospira Inc., Lake Forest, IL.

^4^Maropitant, Zoetis LLC, Kalamazoo, MI.

^5^Trazodone, Major Pharmaceuticals, Livonia, MI.

^6^Calcium gluconate, Fresenius Kabi, Lake Zurich, IL.

^7^Dextrose, MWI, Boise, ID.

^8^Humulin R, Lilly USA LLC, Indianapolis, IN.

^9^Monocryl, Ethicon, Johnson and Johnson, San Lorenzo.

^10^4.7 French 20 cm ureteral stent, Infiniti Medical, Palo Alto, CA.

^11^Jackson-Pratt, Covidien LLC, Mansfield, MA.

^12^Foley urinary catheter, Cook Medical, Bloomington, IN.

^13^7 French triple lumen jugular catheter, Arrow International Inc, Redding, PA.

^14^Fentanyl citrate, Hospira Inc., Lake Forest, IL.

^15^Ketamine, MWI, Boise, ID.

^16^Lidocaine, VetOne®, Las Vegas, NV.

^17^Ondansetron, Accord Healthcare Inc, Durham, NC.

^18^Cisapride (Compounded), Iowa State University College of Veterinary Medicine, Ames, IA.

^19^Gabapentin, Actavis Elizabeth LLC, Elizabeth, NJ.

^20^Pantoprazole, West-ward, Eatontown, NJ.

^21^Magnesium sulfate, Fresenius Kabi, Lake Zurich, IL.

^22^Enrofloxacin (Baytril® injectable), Bayer Healthcare LLC, Shawnee Mission, KS.

^23^Red Rubber catheter, Covidien LLC, Mansfield, MA.

^24^Norepinephrine, Baxter Healthcare, Deerfield, IL.

^25^Chloramphenicol, Bimeda Inc, Le Sueruer, MN.

^26^SMZ–TMP, Rising Health LLC, Saddle Brook, NJ.

^27^French 120 cm basket device, Cook Medical, Bloomington, IN.
